# Neutrophil-to-albumin ratio mediates the association between Life’s Crucial 9 and chronic obstructive pulmonary disease

**DOI:** 10.3389/fmed.2025.1610945

**Published:** 2025-06-23

**Authors:** Jing Feng, Hongyang Gong

**Affiliations:** ^1^Department of Geriatrics, Chengdu Sixth People’s Hospital, Chengdu, China; ^2^Department of Physiology, College of Medicine, Chosun University, Gwangju, Republic of Korea

**Keywords:** Life’s Crucial 9, chronic obstructive pulmonary disease, neutrophil-to-albumin ratio, NHANES, mediation analysis

## Abstract

**Background:**

Chronic obstructive pulmonary disease (COPD) is a progressive respiratory disorder characterized by persistent airflow limitation and chronic airway inflammation. Life’s Crucial 9 (LC9) is a comprehensive tool for evaluating cardiovascular and metabolic health. The neutrophil-to-albumin ratio (NPAR) has been proposed as a novel inflammation-nutrition biomarker. This study aimed to elucidate the association between LC9 scores and the prevalence of COPD while also assessing the potential mediating role of NPAR.

**Methods:**

A cross-sectional analysis was conducted using data from 25,634 U.S. participants in the National Health and Nutrition Examination Survey (NHANES) from 2005 to 2018. Multivariable logistic regression, stratified subgroup analyses, and restricted cubic spline (RCS) models were employed to evaluate the association between LC9 and COPD.

**Results:**

Among the 25,634 participants, 1,248 reported a history of COPD. After adjusting for multiple covariates, each 10-unit increase in the LC9 score was associated with a 28% lower odds of COPD (OR = 0.72, 95% CI: 0.67–0.77), whereas each one-unit increase in NPAR was associated with a 6% higher odds of COPD (OR = 1.06, 95% CI: 1.03–1.10). Similar trends were observed when LC9 and NPAR were categorized into different levels (P for trend < 0.05). RCS analysis revealed a linear inverse relationship between LC9 scores and COPD prevalence. Mediation analysis indicated that NPAR accounted for 4.84% of the association between LC9 and COPD (*p* < 0.001).

**Conclusion:**

Higher LC9 scores were associated with a reduced risk of COPD, with NPAR acting as a significant mediator in this relationship. These findings highlight the potential value of optimizing cardiovascular health in COPD prevention strategies and underscore the importance of controlling inflammation and improving nutritional status. Further prospective studies are warranted to validate these preliminary findings.

## Introduction

Chronic obstructive pulmonary disease (COPD) is a progressive respiratory disorder characterized by persistent airflow limitation, chronic inflammation, and irreversible damage to lung tissue (1, 2). Globally, COPD represents the third leading cause of death, accounting for 3.3 million deaths in 2019 (3), with its prevalence and disease burden continuing to rise, particularly in low- and middle-income countries (4). In addition to its high mortality rate, COPD imposes a substantial socioeconomic burden due to frequent hospitalizations, long-term treatment requirements, and reduced quality of life. Given its chronic and irreversible nature, identifying modifiable risk factors is essential for early prevention and slowing disease progression, ultimately reducing its societal and public health impact.

In recent years, increasing evidence has highlighted a strong link between COPD and cardiovascular health (5, 6). Studies have demonstrated that individuals with poor cardiovascular health are at a significantly elevated risk of developing COPD (7), with systemic inflammation and oxidative stress potentially serving as shared pathogenic mechanisms (8–10). In this context, the American Heart Association (AHA) introduced Life’s Essential 8 (LE8) (11), along with an expanded version, Life’s Crucial 9 (LC9) (12), as comprehensive tools for assessing individual cardiovascular and metabolic health. The LC9 builds upon the LE8 by incorporating psychological well-being, providing a more holistic and scientifically robust framework. LC9 has been applied in studies related to overactive bladder syndrome (13, 14) and infertility (15). Although some studies have preliminarily investigated the direct relationship between LC9 and COPD risk, the potential biological pathways underlying this association remain unclear (16, 17).

Notably, both inflammation and nutritional status are critical determinants of cardiovascular health (18) and key contributors to the pathogenesis of COPD (19). Unhealthy dietary patterns, physical inactivity, and metabolic dysfunction may exacerbate systemic inflammation and accelerate pulmonary function decline. Previous studies have suggested that several inflammation- and nutrition-related biomarkers are closely associated with both cardiovascular and respiratory diseases (19, 20). Among these markers, the neutrophil-to-albumin ratio (NPAR) has recently emerged as a novel indicator of systemic inflammation and nutritional status (21), attracting growing attention. NPAR has been shown to predict adverse outcomes in various cardiopulmonary conditions (22, 23), and elevated NPAR levels have been shown to predict COPD mortality risk (24). Neutrophils indicate systemic inflammation, which contributes to atherosclerosis, endothelial dysfunction, and plays a central role in airway inflammation and lung tissue damage seen in COPD. Albumin, on the other hand, reflects nutritional status and decreases during inflammation. Low albumin levels are linked to poor outcomes in both cardiovascular conditions and COPD (25). By combining these two indicators, NPAR serves as a comprehensive measure of inflammatory and nutritional status. This makes it a biologically plausible mediator between LC9, which reflects cardiovascular health, and the development of COPD. Therefore, exploring NPAR as a potential link can help clarify how systemic processes connect cardiovascular and pulmonary health.

Despite evidence supporting the individual associations of LC9 and NPAR with COPD, no prior study has elucidated whether LC9 may influence COPD risk via modulation of NPAR levels. Conceptually, individuals with better cardiovascular and metabolic health (as captured by higher LC9 scores) are more likely to maintain lower systemic inflammation and better nutritional status—factors directly reflected by a reduced NPAR. In turn, a lower NPAR may attenuate inflammatory-driven lung tissue damage and functional decline, thereby reducing COPD risk. This potential mediating pathway offers a biologically plausible mechanism linking cardiovascular health to pulmonary disease development but has not yet been empirically tested. Therefore, building upon prior research, this study aims to investigate the potential mediating effect of NPAR in the association between LC9 and COPD using data from the 2005–2018 National Health and Nutrition Examination Survey (NHANES). Through mediation analysis, we seek to uncover whether improved cardiovascular health may reduce COPD prevalence partly by lowering systemic inflammation and improving nutritional status, as captured by NPAR. This novel analytical approach may provide new mechanistic insights into the complex interplay among cardiovascular health, inflammation, nutrition, and respiratory disease.

## Methods

### Study participants

The National Health and Nutrition Examination Survey (NHANES) is a complex, multistage probability sampling program conducted by the National Center for Health Statistics (NCHS). It collects comprehensive data on demographic characteristics, health indicators, and dietary patterns of the U.S. population. The primary aim of NHANES is to assess and monitor the nutritional and health status of individuals across all age groups in the United States. All NHANES protocols were approved by the NCHS Research Ethics Review Board, and written informed consent was obtained from all participants. The present study utilized publicly available data and adhered to the STROBE guidelines for cross-sectional studies (26), thus exempting it from further institutional ethical review. Detailed documentation on the NHANES sampling design, methodology, and ethical considerations is available from the official CDC and NCHS websites.^1^

**Figure 1:**
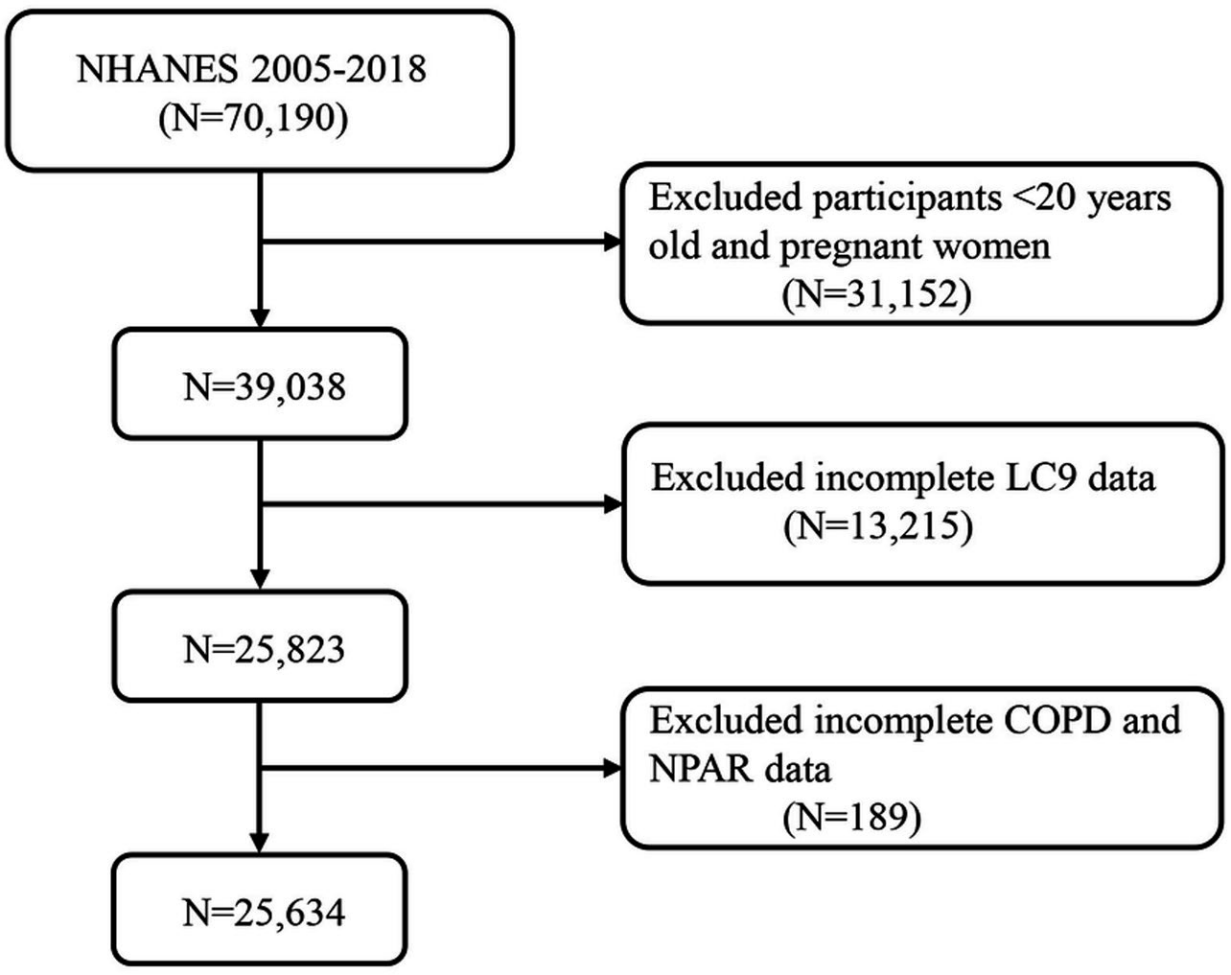
A flow diagram of eligible participant selection in the National Health and Nutrition Examination Survey. LC9, Life’s Crucial 9; NPAR, neutrophil-to-albumin ratio; COPD, chronic obstructive pulmonary disease.

A rigorous participant selection process was applied to ensure data integrity and the reliability of the findings. Data from NHANES 2005–2018 were utilized. The participant flow is illustrated in Figure 1. Initially, 70,190 individuals were considered. Participants aged under 20 years and pregnant women (*N* = 31,152) were excluded, leaving 39,038 eligible individuals. Subsequently, 13,215 participants with incomplete LC9 data were removed, resulting in a sample of 25,823 individuals. Finally, 189 participants with missing data on NPAR or COPD-related variables were excluded, yielding a final analytical sample of 25,634 participants. This careful screening ensured the completeness and representativeness of the study population.

### COPD assessment

Consistent with previous research (27), COPD was defined using one or more of the following criteria: (1) post-bronchodilator FEV1/FVC ≤ 0.70; (2) the presence of emphysema or self-reported COPD; or (3) participants aged ≥ 40 years with a history of chronic bronchitis or smoking and currently using COPD-related medications, including mast cell stabilizers, inhaled corticosteroids, selective phosphodiesterase-4 inhibitors, or leukotriene modifiers.

### Definition of neutrophil-to-albumin ratio (NPAR)

Data used to calculate the NPAR were obtained from the complete blood count (CBC) panel in the NHANES database. CBC parameters were measured using the Beckman Coulter method (28). Detailed information regarding laboratory methods, quality control, and data handling procedures can be accessed via the NHANES website: https://wwwn.cdc.gov/Nchs/Data/Nhanes/Public/2017/DataFiles/CBC_J.htm. Albumin levels were assessed using standard NHANES biochemical protocols, specifically via a digital endpoint method with dual-wavelength detection (29). The NPAR was calculated using the following formula: NPAR = (Neutrophil percentage [%] × 100) / Albumin (g/dL) (28).

### Definition of Life’s Crucial 9 (LC9)

LC9 integrates nine key health metrics, categorized into behavioral components (diet quality, physical activity, smoking avoidance, and sleep health) and physiological components (weight management, blood lipids, glucose control, blood pressure regulation, and mental health) (13, 14). The specific NHANES-based scoring methodology is detailed in Supplementary Table S1. Each component was assigned a score ranging from 0 to 100, and the overall LC9 score was calculated as the average of the nine components. Diet quality was assessed using the 2015 Healthy Eating Index (HEI-2015) (15), with detailed scoring components presented in Supplementary Table S2. Behavioral health data were collected through standardized questionnaires, whereas clinical measurements, including anthropometry, cardiovascular parameters, and metabolic markers, were obtained by trained personnel from the NHANES database (see text footnote 1).

### Covariables

Covariates included in this study were age, sex, race/ethnicity, marital status, education level, poverty income ratio (PIR), hypertension, diabetes, and hyperlipidemia. Detailed descriptions of these covariates are provided in Supplementary Table S3.

### Statistical analysis

The R software environment was used for statistical processing (version 4.3.1). We used suitable sampling weights in all analyses to guarantee population-level inference and take into consideration the intricate sampling design of NHANES. Using weighted t-tests for between-group comparisons, descriptive statistics displayed continuous variables as weighted averages with standard deviations. Weighted frequencies with percentages were used to express categorical variables, and weighted chi-square techniques were used to evaluate differences between groups.

The multivariable logistic regression models (1) unadjusted, (2) demographically adjusted (adjusting for age, sex, education, marital status, poverty ratio, and race/ethnicity), and (3) fully adjusted (adding clinical covariates such as hypertension, diabetes, and hyperlipidemia) were used to investigate LC9-COPD and NPAR-COPD relationships. To assess possible non-linear relationships between variables and outcomes, we used restricted cubic spline modeling. The consistency of identified relationships across population strata was investigated by thorough subgroup analyses.

To assess NPAR’s potential mediating function in the LC9-COPD relationship, formal mediation analysis was performed to quantify direct effects, indirect effects, and total effects of LC9 on COPD development through NPAR pathways. The mediation proportion was mathematically derived as (indirect effect / [indirect effect + direct effect]) × 100% (27). All mediation effect evaluations were executed utilizing the specialized “mediation” package within the R statistical environment (27). Statistical significance was established at a two-sided probability threshold of *p* < 0.05.

## Result

### Baseline characteristics

Table 1 presents the baseline characteristics of 25,634 participants, including 1,248 individuals with COPD and 24,386 without COPD. After applying sample weights, the findings were extrapolated to represent approximately 120,660,843 U.S. adults, among whom an estimated 5,809,388 had COPD, corresponding to a national prevalence of 4.8%.

**Table 1:** Baseline characteristics of all participants were stratified by COPD, weighted.

Characteristic	Overall, *N* = 120,660,843 (100%)	Non-COPD, *N* = 114,851,454 (95.2%)	COPD, *N* = 5,809,388 (4.8%)	*p* Value
No. of participants in the sample	25,634	24,386	1,248	**-**
Age (%)				**<0.001**
20–40	42,175,998 (35%)	41,859,837 (36%)	316,161 (5.4%)	
41–60	45,482,817 (38%)	43,152,400 (38%)	2,330,417 (40%)	
>60	33,002,028 (27%)	29,839,218 (26%)	3,162,810 (54%)	
Sex (%)				0.276
Female	62,042,783 (51%)	59,195,517 (52%)	2,847,266 (49%)	
Male	58,618,060 (49%)	55,655,938 (48%)	2,962,122 (51%)	
Race (%)				**<0.001**
Non-Hispanic White	85,637,276 (71%)	80,764,126 (70%)	4,873,151 (84%)	
Non-Hispanic Black	12,273,589 (10%)	11,919,916 (10%)	353,674 (6.1%)	
Other	13,871,985 (11%)	13,385,965 (12%)	486,020 (8.4%)	
Mexican American	8,877,992 (7.4%)	8,781,448 (7.6%)	96,544 (1.7%)	
Married/live with partner (%)				0.570
No	42,987,128 (36%)	40,973,888 (36%)	2,013,240 (35%)	
Yes	77,641,283 (64%)	73,845,135 (64%)	3,796,148 (65%)	
Education level (%)				**0.001**
Below high school	16,821,258 (14%)	15,741,070 (14%)	1,080,188 (19%)	
High School or above	103,802,597 (86%)	99,073,397 (86%)	4,729,200 (81%)	
PIR (%)				**0.004**
Poor	21,961,758 (19%)	20,685,929 (19%)	1,275,830 (24%)	
Not poor	91,404,896 (81%)	87,268,735 (81%)	4,136,161 (76%)	
Hypertension (%)				**<0.001**
No	73,643,152 (61%)	71,157,478 (62%)	2,485,674 (43%)	
Yes	47,017,690 (39%)	43,693,976 (38%)	3,323,714 (57%)	
Diabetes (%)				**<0.001**
No	105,337,328 (87%)	100,827,270 (88%)	4,510,058 (78%)	
Yes	15,323,515 (13%)	14,024,184 (12%)	1,299,330 (22%)	
Hyperlipidemia (%)				**<0.001**
No	34,172,193 (28%)	33,192,788 (29%)	979,406 (17%)	
Yes	86,488,649 (72%)	81,658,667 (71%)	4,829,982 (83%)	
Mean LC9 score [mean (SE)]	70.44 (13.61)	70.82 (13.49)	62.92 (13.74)	**<0.001**
LC9, Tertile (%)				**<0.001**
T1	39,075,080 (32%)	36,032,687 (31%)	3,042,393 (52%)	
T2	40,813,205 (34%)	39,048,862 (34%)	1,764,344 (30%)	
T3	40,772,557 (34%)	39,769,906 (35%)	1,002,651 (17%)	
Mean psychological health score [mean (SE)]	89.26 (23.27)	89.52 (22.96)	84.10 (28.33)	**<0.001**
Mean HEI-2015 diet score [mean (SE)]	39.52 (31.43)	39.55 (31.48)	38.82 (30.36)	0.625
Mean physical activity score [mean (SE)]	71.71 (41.06)	72.24 (40.74)	61.20 (45.64)	**<0.001**
Mean tobacco exposure score [mean (SE)]	71.32 (38.60)	72.38 (38.27)	50.36 (39.20)	**<0.001**
Mean sleep health score [mean (SE)]	83.45 (24.21)	83.66 (24.00)	79.23 (27.64)	**<0.001**
Mean body mass index score (mean (SE))	60.55 (33.51)	60.68 (33.49)	58.10 (33.68)	0.052
Mean blood lipid score[mean (SE)]	63.92 (30.32)	64.21 (30.33)	58.32 (29.61)	**<0.001**
Mean blood glucose score [mean (SE)]	85.61 (24.20)	86.08 (23.93)	76.23 (27.49)	**<0.001**
Mean blood pressure score [mean (SE)]	68.61 (31.13)	69.05 (31.09)	59.97 (30.57)	**<0.001**
NPAR [mean (SE)]	13.75 (2.46)	13.71 (2.44)	14.47 (2.70)	**<0.001**
NPAR, Tertile (%)				<0.001
T1	40,253,840 (33%)	38,805,903 (34%)	1,447,937 (25%)	
T2	40,167,876 (33%)	38,492,849 (34%)	1,675,026 (29%)	
T3	40,239,127 (33%)	37,552,702 (33%)	2,686,425 (46%)	

Mean (SE) for continuous variables: the P value was calculated by the weighted Students *T*-test. Percentages (weighted N, %) for categorical variables: the P value was calculated by the weighted chi-square test. Abbreviation: LC9, Life’s Crucial 9; NPAR, neutrophil-to-albumin ratio; PIR, poverty income ratio; COPD, chronic obstructive pulmonary disease. The bold values are less than 0.05.

Significant differences were observed between the COPD and non-COPD groups in terms of age distribution, race/ethnicity, educational level, family income, hypertension, diabetes, and hyperlipidemia (all *p* < 0.05). Participants with COPD were generally older, more likely to be White, and had higher rates of hypertension, diabetes, and hyperlipidemia. Moreover, the LC9 scores were significantly lower in the COPD group compared to the non-COPD group (*p* < 0.001), while NPAR values were significantly higher (*p* < 0.001). A comprehensive summary of the characteristics is shown in Table 1.

### Association between LC9, NPAR, and COPD

As shown in Table 2, we constructed three progressively adjusted multivariable models to examine the association between LC9 and COPD. Across all models, LC9 was consistently and significantly inversely associated with COPD (all *p* < 0.05). In the fully adjusted model, controlling for all potential confounders, every 10-unit increase in LC9 was associated with a 28% lower likelihood of having COPD (OR = 0.72, 95% CI: 0.67–0.77, *p* < 0.001). In the sensitivity analysis using LC9 tertiles, individuals in the highest tertile (T3) had a 56% lower prevalence of COPD compared to those in the lowest tertile (OR = 0.44, 95% CI: 0.34–0.58, *p* < 0.001).

**Table 2:** Association between LC9, NPAR, and COPD, NHANES 2005–2018.

Characteristics	Model 1 [OR (95% CI)]	*p-value*	Model 2 [OR (95% CI)]	*p-value*	Model 3 [OR (95% CI)]	*p-value*
LC9 - COPD						
Continuous (per 10 scores)	0.67(0.63,0.71)	<0.001	0.74(0.69, 0.78)	<0.001	0.72(0.67, 0.77)	<0.001
Tertile						
T1	1 (ref.)		1 (ref.)		1 (ref.)	
T2	0.54(0.44,0.65)	<0.001	0.65(0.54, 0.79)	<0.001	0.65(0.54, 0.80)	<0.001
T3	0.30(0.24,0.37)	<0.001	0.45(0.35, 0.57)	<0.001	0.44(0.34, 0.58)	<0.001
*P* for trend	<0.001		<0.001		0.004	
NPAR - COPD						
Continuous	1.13(1.10,1.16)	<0.001	1.07(1.03, 1.10)	<0.001	1.06(1.03, 1.10)	<0.001
Tertile						
T1	1 (ref.)		1 (ref.)		1 (ref.)	
T2	1.17(0.95,1.43)	0.140	0.99(0.79, 1.23)	0.900	0.98(0.78, 1.22)	0.840
T3	1.92(1.57,2.34)	<0.001	1.38(1.11, 1.70)	0.004	1.35(1.09, 1.66)	0.010
*P* for trend	<0.001		0.002		0.004	

Model 1: no covariates were adjusted. Model 2: age, sex, education level, marital, PIR, and race were adjusted. Model 3: age, sex, education level, marital, PIR, race, hypertension, diabetes, and hyperlipidemia were adjusted. LC9, Life’s Crucial 9; NPAR, neutrophil-to-albumin ratio; PIR, poverty income ratio; COPD, chronic obstructive pulmonary disease; OR, odds ratio; CI, confidence interval.

Regarding NPAR, higher levels were positively associated with COPD risk. Specifically, each unit increase in NPAR was associated with a 6% increase in COPD prevalence (OR = 1.06, 95% CI: 1.03–1.10, *p* < 0.001).

**Figure 2:**
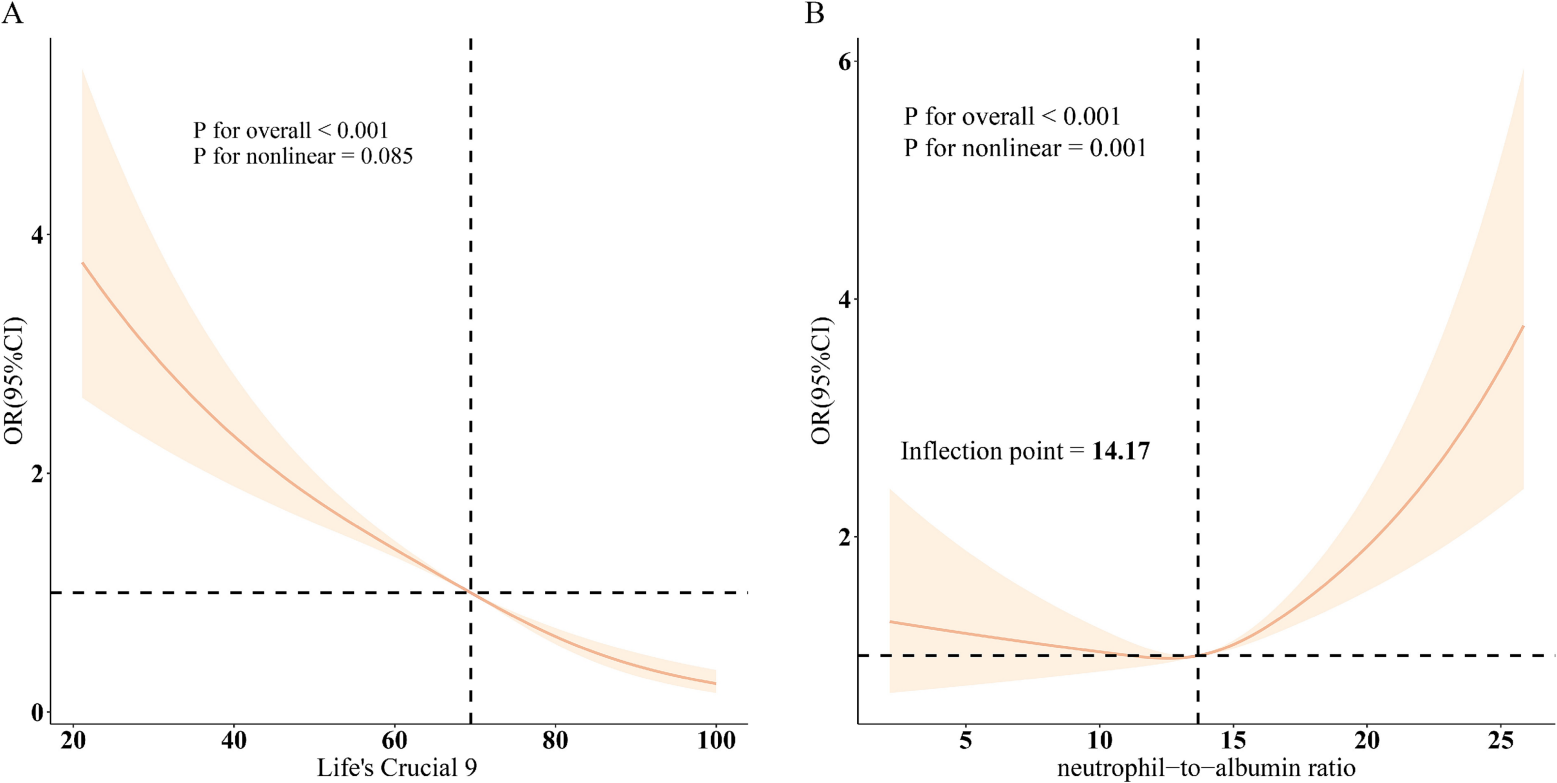
Dose–response relationships between LC9, NPAR, and COPD. **(A)**, LC9 - COPD; **(B)**, NPAR - COPD. OR (solid lines) and 95% confidence levels (shaded areas) were adjusted for age, sex, education level, marital, PIR, race, hypertension, diabetes, and hyperlipidemia.

Restricted cubic spline models (Figure 2) confirmed a linear inverse relationship between LC9 and COPD risk after adjusting for all covariates (overall *p* < 0.001; nonlinearity *p* > 0.05). In contrast, the association between NPAR and COPD was nonlinear, with an inflection point at 14.17. Threshold effect analysis (Table 3) revealed that NPAR was not significantly associated with COPD when levels were below 14.17 (OR = 1.01, 95% CI: 0.97–1.06) but showed a significant positive association when levels exceeded 14.17 (OR = 1.13, 95% CI: 1.09–1.17).

**Table 3:** Threshold effect analysis of the relationship between NPAR and COPD.

Adjusted	OR (95%CI), *p* value
Inflection point	14.17
NPAR<14.17	1.01 (0.97, 1.06), 0.588
NPAR≥14.17	1.13 (1.09, 1.17), <0.001
P for likelihood ratio test	0.002

Adjusted for age, sex, education level, marital, PIR, race, hypertension, diabetes, and hyperlipidemia.

**Figure 3:**
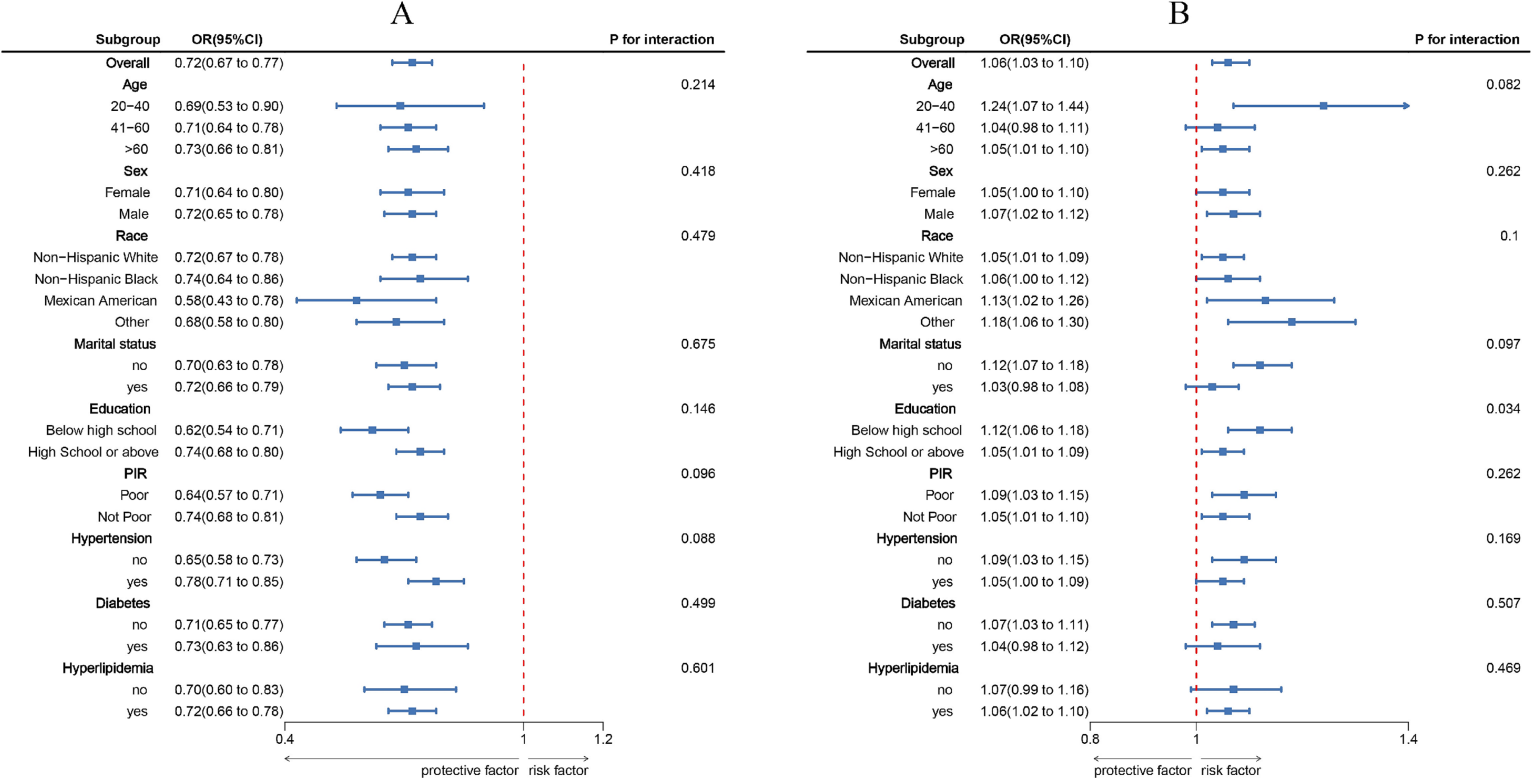
Subgroup analysis between LC9, NPAR, and COPD. **(A)**, LC9 - COPD; **(B)**, NPAR - COPD. ORs were calculated per 10-unit increase in LC9, and each standard deviation increased in NPAR. Analyses were adjusted for age, sex, education level, marital, PIR, race, hypertension, diabetes, and hyperlipidemia.

Subgroup analyses stratified by age, sex, race/ethnicity, marital status, educational level, PIR, hypertension, diabetes, and hyperlipidemia (Figure 3) showed that the inverse association between LC9 and COPD, as well as the positive association between NPAR and COPD, remained consistent across all subgroups. No significant interactions were observed (all p for interaction > 0.05).

### Mediation effect

**Figure 4:**
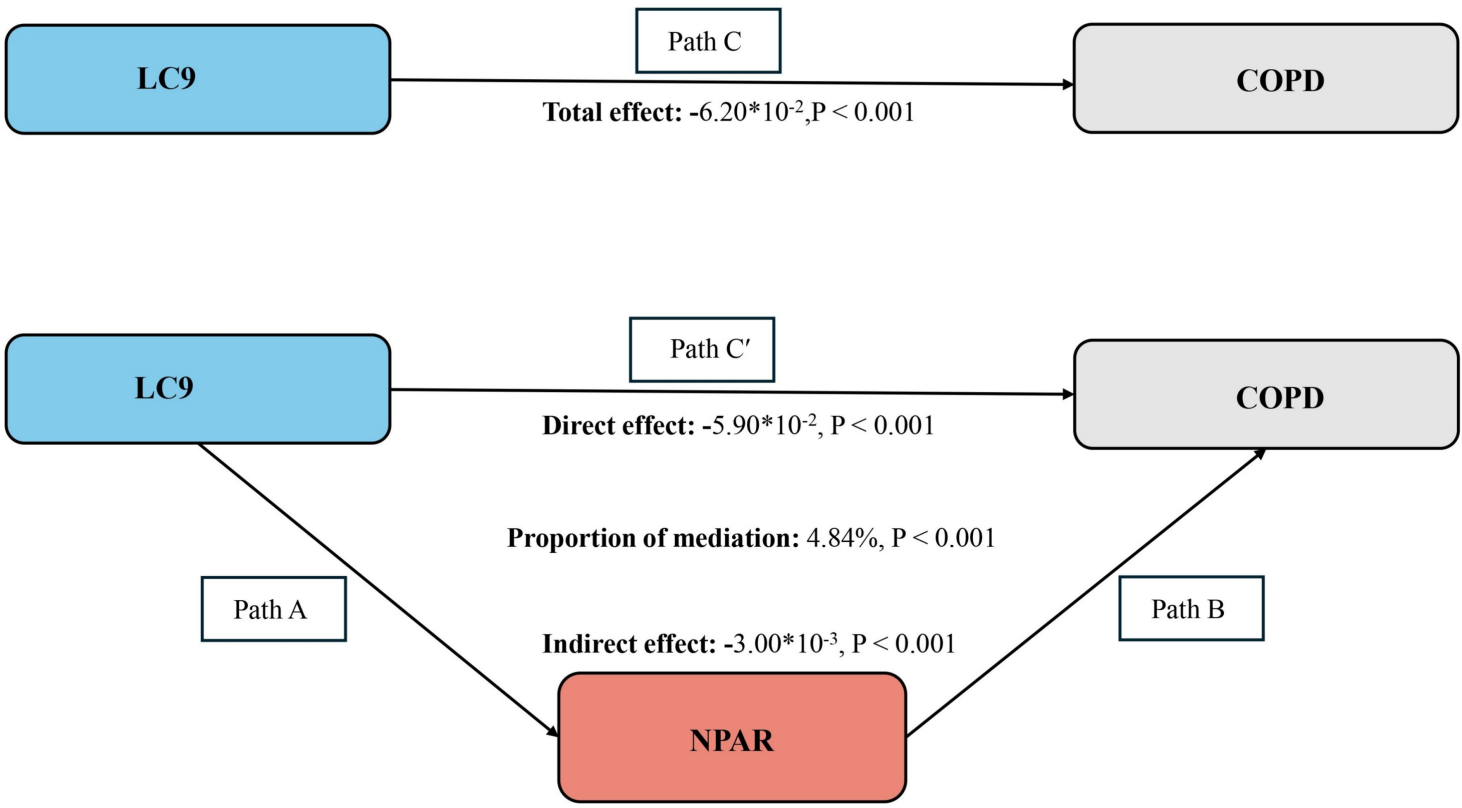
Schematic diagram of the mediation effect analysis. Path C indicates the total effect; path C′ indicates the direct effect. The indirect effect is estimated as the multiplication of paths A and B (path A*B). The mediated proportion is calculated as indirect effect/ (indirect effect + direct effect) × 100%. Abbreviation: LC9, Life’s Crucial 9; NPAR, neutrophil-to-albumin ratio; COPD, chronic obstructive pulmonary disease. Analyses were adjusted for age, sex, education level, marital, PIR, race, hypertension, diabetes, and hyperlipidemia.

As illustrated in Figure 4, a mediation analysis was conducted with LC9 as the independent variable, COPD as the dependent variable, and NPAR as the mediator. After adjusting for all covariates, Table 4 indicates a significant negative association between LC9 and NPAR (*β* = −0.27, 95% CI: −0.31 to −0.24). Further analysis confirmed the mediation role of NPAR (indirect effect = −3.00 × 10^−3^, *p* < 0.001; direct effect = −5.90 × 10^−2^, *p* < 0.001). The proportion of the effect of LC9 on COPD mediated by NPAR was 4.84% (*p* < 0.001). These results suggest that NPAR partially mediates the relationship between LC9 and COPD.

**Table 4:** Multivariate linear regression of LC9 and NPAR.

	β	95%CI	*p*-value
LC9 - NPAR	−0.27	(−0.31, −0.24)	<0.001

Adjusted for age, sex, education level, marital, PIR, race, hypertension, diabetes, and hyperlipidemia.

## Discussion

In this nationally representative study of U.S. adults, a significant association was observed between Life’s Crucial 9 (LC9), the neutrophil-to-albumin ratio (NPAR), and chronic obstructive pulmonary disease (COPD). After full adjustment for potential confounders, each 10-point increase in LC9 score was associated with a 28% reduction in COPD prevalence. Conversely, each 1-unit increase in NPAR was associated with a 1.06-fold increase in COPD prevalence. Mediation analysis further revealed that NPAR partially mediated the relationship between LC9 and COPD, suggesting that LC9 may influence COPD risk in part by improving systemic inflammation, as reflected by NPAR levels.

By capturing multiple dimensions of cardiovascular health, LC9 offers a comprehensive evaluation of overall health status, potentially enabling a more accurate assessment of COPD risk. These findings provide a theoretical foundation for further exploration of cardiopulmonary interactions and underscore the importance of cardiovascular health management in the prevention of chronic respiratory diseases. Moreover, the results may inform public health policy, supporting the implementation of integrative interventions aimed at reducing COPD prevalence and improving quality of life. Future research is warranted to investigate the broader applicability of LC9 in the early detection and management of other chronic diseases, thereby advancing the paradigm of holistic health management.

The favorable cardiovascular health behaviors and factors reflected in higher LC9 scores may mitigate the development and progression of COPD through various biological pathways. One hallmark of COPD is chronic and localized hypoxia, which promotes the release of pro-inflammatory cytokines such as IL-6, TNF-*α*, and IL-1β (30, 31), thereby triggering systemic inflammation and an “inflammatory storm.” Several LC9 components—such as a healthy diet and optimal glycemic and lipid control—may reduce systemic inflammation by inhibiting oxidative stress and suppressing pro-inflammatory cytokine production (32). Furthermore, individuals with higher LC9 scores often exhibit improved vascular function and tissue perfusion, which may enhance pulmonary oxygenation, thus alleviating hypoxia-induced tissue damage and immune activation. A healthy lifestyle may also influence the secretory functions of alveolar epithelial and immune cells (33), reducing the excessive release of extracellular matrix-degrading enzymes and mucins, thereby slowing airway remodeling and obstruction.

Additionally, the inverse association between LC9 and COPD may be mediated through the regulation of both programmed and non-programmed cell death pathways. Recent studies have identified significant dysregulation of ferroptosis (34), pyroptosis (35), apoptosis (36), and cuproptosis (37) in the lung tissues of COPD patients. These forms of cell death are closely related to oxidative stress, metal ion imbalance, and chronic inflammation. Beneficial health behaviors encompassed in LC9, such as antioxidant-rich diets and physical activity, may help maintain redox homeostasis and mitochondrial function, thereby suppressing lipid peroxidation and ferroptosis (38, 39).

In the present study, NPAR was found to partially mediate the association between LC9 and COPD, suggesting that systemic inflammation may serve as a key biological pathway linking cardiovascular health and pulmonary outcomes. LC9, as a multidimensional index of cardiovascular health, incorporates both behavioral and physiological factors—including smoking status, physical activity, diet quality, body weight, blood pressure, blood lipids, blood glucose, sleep, and mental health—all of which are closely linked to systemic inflammation. NPAR, an easily obtainable biomarker, reflects systemic inflammatory and nutritional status (40, 41). In the pathogenesis of COPD, inflammation plays a central role in airway remodeling, lung tissue destruction, and functional decline (42, 43). Lower LC9 scores may reflect unhealthy lifestyle patterns and metabolic disturbances, contributing to elevated NPAR levels and the amplification of both airway and systemic inflammation, thereby promoting COPD onset and progression. These findings support a mediating role of inflammation in the LC9-COPD association and suggest that optimizing LC9 components and reducing systemic inflammation may be effective strategies for COPD prevention and management.

This study is the first to systematically explore the interrelationships and mediating role of LC9, NPAR, and COPD, contributing to the theoretical foundation for LC9 application in respiratory disease research and offering new evidence for the involvement of NPAR in COPD pathogenesis. The study leveraged data from the National Health and Nutrition Examination Survey (NHANES), which features a large, nationally representative sample, thereby enhancing the generalizability and scientific robustness of the findings. Comprehensive statistical adjustments were made for a range of potential confounders, including age, sex, race/ethnicity, and socioeconomic status, using multivariable regression models to improve the reliability and validity of the results. Furthermore, subgroup analyses were conducted to verify the consistency and stability of the observed associations across various population strata, strengthening the credibility and translational value of the findings.

Several limitations of this study warrant consideration. First, the cross-sectional design of NHANES precludes the establishment of causal relationships among LC9, NPAR, and COPD. Although multiple sociodemographic, lifestyle, and health-related covariates were adjusted for, the influence of unmeasured or residual confounding—such as environmental exposures and genetic susceptibility—cannot be entirely ruled out. Second, reliance on self-reported data in NHANES may introduce information bias. Additionally, NPAR was only measured during specific survey cycles, potentially limiting sample size and representativeness. Furthermore, the diagnosis of COPD was based on a combination of self-reported physician diagnosis, post-bronchodilator spirometry results (FEV1/FVC ≤ 0.70), a history of chronic bronchitis or emphysema, smoking history, and the use of COPD-related medications. Due to the constraints of the NHANES database, objective confirmation of COPD for all participants—such as universal availability of spirometry data—was not feasible. As a result, some individuals may have been classified as having COPD solely based on self-reported information without corroboration by objective diagnostic measures. This may have led to misclassification bias and impacted the specificity of COPD case identification. Second, medication use, and medical history were self-reported, which could have introduced recall bias. Despite these limitations, our definition of COPD aligns with criteria commonly used in previous NHANES-based studies and allows for comparability of findings. Future research with access to more robust and objective diagnostic indicators is warranted to further validate these associations.

Finally, due to the inherent constraints of the NHANES database, we were unable to obtain detailed information regarding participants’ recent or chronic infections, hematological diseases, serious liver diseases, recent major trauma, or major surgery. These conditions may significantly affect neutrophil and albumin levels, potentially confounding the association between the neutrophil-to-albumin ratio and chronic obstructive pulmonary disease. As a result, we could not exclude such individuals from our analysis. Future studies with more comprehensive clinical data collection are warranted to address and minimize these potential confounding factors.

## Conclusion

In conclusion, our study demonstrates a significant inverse association between LC9 and COPD, with NPAR playing a partial mediating role. These findings highlight the potential link between cardiovascular health and COPD, underscoring the critical role of inflammation regulation and nutritional management in this relationship. This study offers a novel perspective for the prevention and management of COPD, suggesting that improving cardiovascular health and adopting comprehensive strategies to control inflammation and optimize nutritional status may help reduce the risk of COPD.

## Data Availability

Publicly available datasets were analyzed in this study. This data can be found here: https://wwwn.cdc.gov/nchs/nhanes/.

## Footnotes

^1^ https://www.cdc.gov/nchs/nhanes/index.htm
